# MEMS-actuated terahertz metamaterials driven by phase-transition materials

**DOI:** 10.1007/s12200-024-00116-4

**Published:** 2024-05-27

**Authors:** Zhixiang Huang, Weipeng Wu, Eric Herrmann, Ke Ma, Zizwe A. Chase, Thomas A. Searles, M. Benjamin Jungfleisch, Xi Wang

**Affiliations:** 1 Department of Materials Science and Engineering, College of Engineering, University of Delaware, Newark, DE 19716 USA; 2 Department of Physics and Astronomy, College of Arts and Sciences, University of Delaware, Newark, DE 19716 USA; 3 Department of Electrical and Computer Engineering, College of Engineering, University of Illinois Chicago, Chicago, IL 60607 USA

**Keywords:** Metamaterials, MEMS, THz, VO_2_, Phase-transition material

## Abstract

The non-ionizing and penetrative characteristics of terahertz (THz) radiation have recently led to its adoption across a variety of applications. To effectively utilize THz radiation, modulators with precise control are imperative. While most recent THz modulators manipulate the amplitude, frequency, or phase of incident THz radiation, considerably less progress has been made toward THz polarization modulation. Conventional methods for polarization control suffer from high driving voltages, restricted modulation depth, and narrow band capabilities, which hinder device performance and broader applications. Consequently, an ideal THz modulator that offers high modulation depth along with ease of processing and operation is required. In this paper, we propose and realize a THz metamaterial comprised of microelectromechanical systems (MEMS) actuated by the phase-transition material vanadium dioxide (VO_2_). Simulation and experimental results of the three-dimensional metamaterials show that by leveraging the unique phase-transition attributes of VO_2_, our THz polarization modulator offers notable advancements over existing designs, including broad operation spectrum, high modulation depth, ease of fabrication, ease of operation condition, and continuous modulation capabilities. These enhanced features make the system a viable candidate for a range of THz applications, including telecommunications, imaging, and radar systems.

**Graphical Abstract:**

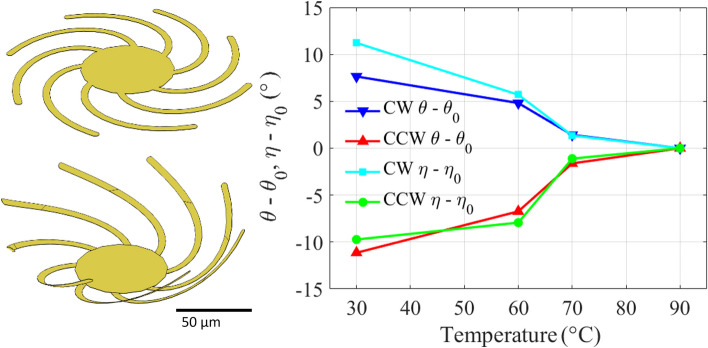

**Supplementary Information:**

The online version contains supplementary material available at 10.1007/s12200-024-00116-4.

## Introduction

Terahertz (THz) radiation, with frequencies ranging from 0.3 to 3 × 10^12^ Hz [1], bridges the gap between the microwave and infrared spectral ranges and can interact with many dielectric materials [2] and intrinsic semiconductors [3], but undergoes large absorption in liquid water [4]. THz technology has been utilized across a diverse range of applications such as imaging [5, 6], security [6], telecommunications [7], and biosensing [8], largely due to its non-ionizing [9] and penetrative characteristics. The effective application of THz radiation requires precise control and manipulation, typically facilitated by THz modulators.

Recently, significant effort has been dedicated to realizing THz modulators, leading to four primary modulation techniques [10]. Carrier concentration modulation employs gating methods or optical pumping to alter an active layer’s optical conductivity, tuning the device’s responsivity to THz waves. These devices have shown outstanding performance in modulation speed and amplitude modulation depth [11–13], but typically involve high gating voltages [12, 14–16]. Liquid crystal modulation utilizes the inherent birefringence of liquid crystals [17–19], but the trade-off between the thickness of the liquid crystal layer and the modulation speed impedes its further development [20, 21]. The phase-transition material vanadium dioxide (VO_2_) introduces a unique THz modulation approach due to its temperature-induced insulator-to-metal transition at around 68°C [22–27]. Metasurfaces integrated with VO_2_ thin films can achieve a large modulation depth with a low operation voltage. However, while conventional VO_2_ devices leverage the optical properties of VO_2_ before and after its phase transition, their design flexibility can be further improved by introducing geometric changes [26, 28–33]. Lastly, microelectromechanical systems (MEMS) can be designed to precisely interact with incident THz waves for effective modulation and tailorable scattered fields [21, 34–40]. Furthermore, the large geometric deformation associated with MEMS enables relatively large modulation by deforming in and out of resonance with the impinging THz wavefront. Even with these advantages, many MEMS are operated by electrostatic forces with operation voltages as high as 300 V, limiting the maximum achievable deformation and resulting modulation depth [41–44].

Typical THz modulators manipulate key parameters of incident light, such as amplitude, frequency, phase, and polarization. Among them, THz pulses with modulated polarization are promising for probing and manipulating materials via crystal lattice vibrations [45], molecular rotation and alignment [46], and spin processing [47–49]. Other key technologies that demand meticulous polarization control include THz telecommunication [50], circular dichroism spectroscopy [51], and THz coherent control of matter [52]. However, despite the recent advances in the field of polarization modulation, there are still some limitations that impede further improvement and broader applications. Many active polarization management systems demand intricate optical stimulus configurations and extended propagation lengths to detect chirality in natural substances, which result in complicated device production [53]. Traditional polarization control techniques are typically restricted in bandwidth and frequency, which exhibit suboptimal efficiency and a restricted modulation depth [19, 20, 54, 55]. Additionally, conventional methods for polarization control require high driving voltages [43], ion-gel control [51], or a sealed gas chamber [39].

Here, we propose a MEMS-based THz polarization modulator that relies on the mechanical deformation driven by the phase transition of VO_2_. Our modulator, fabricated by one-step photolithography, can achieve active tuning of the polarization of THz waves with ease of operation near room temperature. The operation wavelength range and modulation depth of polarization are comparable with previous reports using high driving voltages [43] and pneumatic forces [39].

## Results

### Dynamically tunable metamaterials

**Fig. 1:**
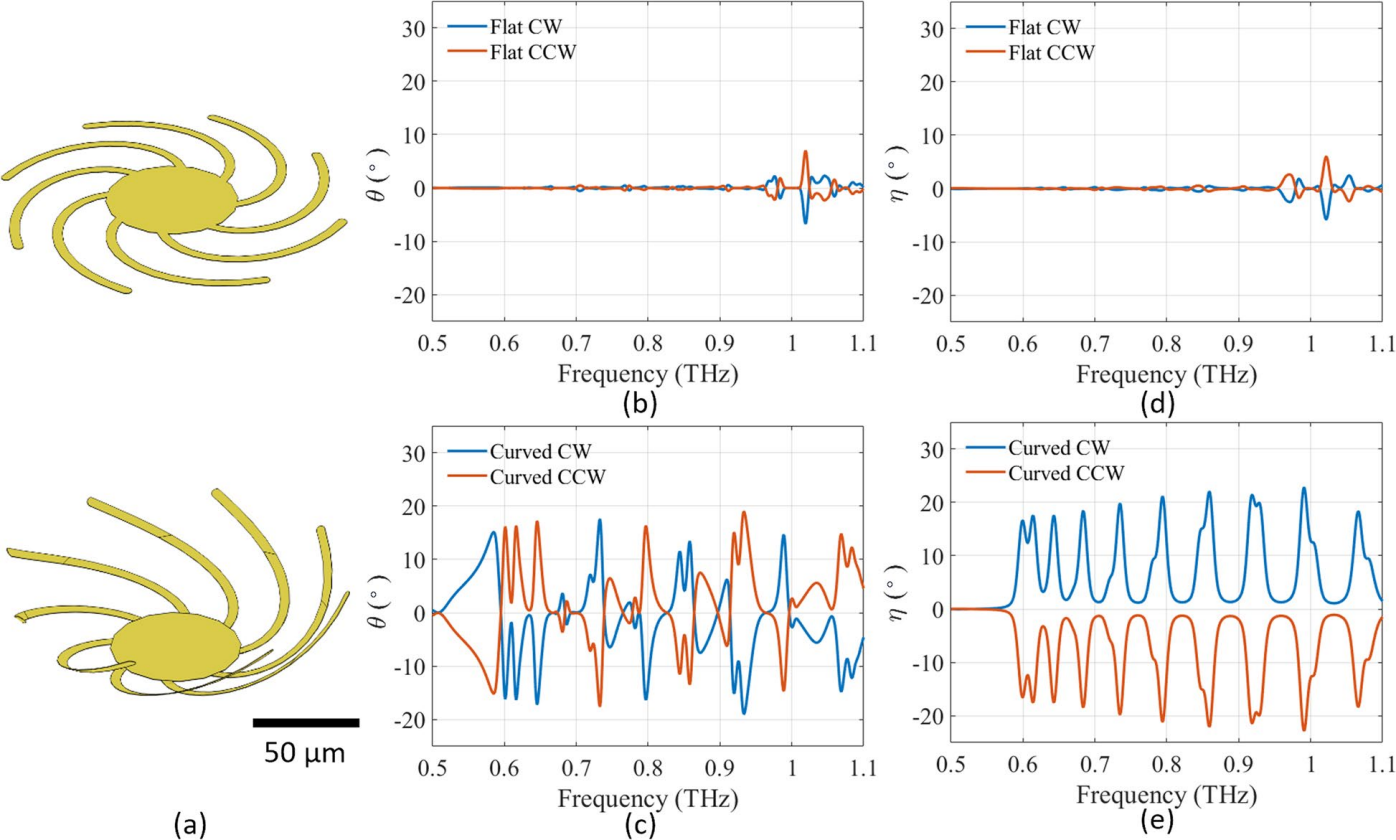
**a** Schematic of counterclockwise spiral cantilevers fully flat (top) and curving up (bottom) (Note: clockwise spiral cantilevers are also made). **b** Simulated azimuth polarization rotation angle *θ* for flat spiral cantilevers. **c** Simulated azimuth polarization rotation angle *θ* for curved spiral cantilevers. **d** Simulated ellipticity angle *η* for flat spiral cantilevers. **e** Simulated ellipticity angle *η* for curved spiral cantilevers

We employ a tunable chiral metasurface to dynamically modulate the polarization of incident THz waves. The metasurface consists of a square lattice of patterned unit cells, with each unit built from eight spiral cantilever arms extending from the center anchor, as shown in Fig. 1a. The constant speed spiral cantilevers are equally spaced at any given angle, and the radial distance from the center of the spiral increases linearly with the angle. The spiral cantilevers are connected to a round center anchor, which is stationary during the actuation. By manipulating the length and curvature of helical structures within 3D meta-atoms, significant circular dichroism (CD) can be realized and customized for various wavelengths. Similar structures have been used to modulate the chirality of infrared light [56, 57] and microwave [58].

Tunable polarization modulation can be achieved by changing the curvature of spiral cantilevers. The polarization modulation performance is evaluated by two key parameters: the polarization azimuth rotation angle *θ* and the ellipticity angle *η*. The polarization azimuth rotation angle describes the relative rotation of the maximum polarization, while the ellipticity angle describes the shape of the polarization ellipse.

The polarization modulation performance of the proposed design in Fig. 1a was simulated using COMSOL Multiphysics as detailed in Supplementary Information. Because substrates in real devices limit the downward bending of cantilevers, we simulated two groups of spiral cantilevers, one with clockwise (CW) spiral cantilevers and the other with counterclockwise (CCW) spiral cantilevers, both approximated as perfect electrical conductors. Our device operates on a principle analogous to that of enantiomers, incorporating spiral cantilever arms to create a chiral metamaterial structure. By changing the curvature of the cantilever arms, they exhibit significant optical activity through the exploitation of first-order spatial dispersion effects. This phenomenon arises from the non-local nature of light-matter interactions, where achieving a pronounced three-dimensional spatial variation within the chiral structures is crucial for amplifying optical activity [59]. For example, when the curvature is zero and spiral cantilevers are flat, the entire unit cell is orthogonal to the THz transmission direction and only alters the polarization and ellipticity of the incident THz by a very small value, as shown in Fig. 1b and d. When the spiral cantilevers bend toward the direction of the incident THz beam and form a 3D structure at certain resonance frequencies, this position-dependent arrival time causes a rotation in the polarization azimuth angle, altering the direction of the electric field vector of the transmitted THz beam. As a result, the angle at which the electric field oscillates relative to the initial polarization direction changes, leading to a rotation in the polarization azimuth angle as in Fig. 1c and ellipticity angle as in Fig. 1e.

After validation of the design by simulations, we fabricated the device and utilized VO_2_ as the activation material without the requirement of a critical operating environment, e.g., either a high operation temperature or a sealed chamber as demonstrated before [39]. As a phase transition material, VO_2_ is notable for its temperature-sensitive electronic and optical properties. VO_2_ undergoes a 0.3% volume shrinkage during its phase transition, making it an excellent actuation material with a high volumetric working density [25]. In comparison with other materials such as thermal expansion substances [60, 61], piezoelectrics [62, 63], and shape memory alloys [64, 65], VO_2_-based actuators demonstrate superior performance. While their volumetric working densities reach up to 7 J/cm^3^, they exhibit a rapid response time in the picosecond range for the insulator-to-metal phase-transition and up to 6 kHz for cycling [66–68].

**Fig. 2:**
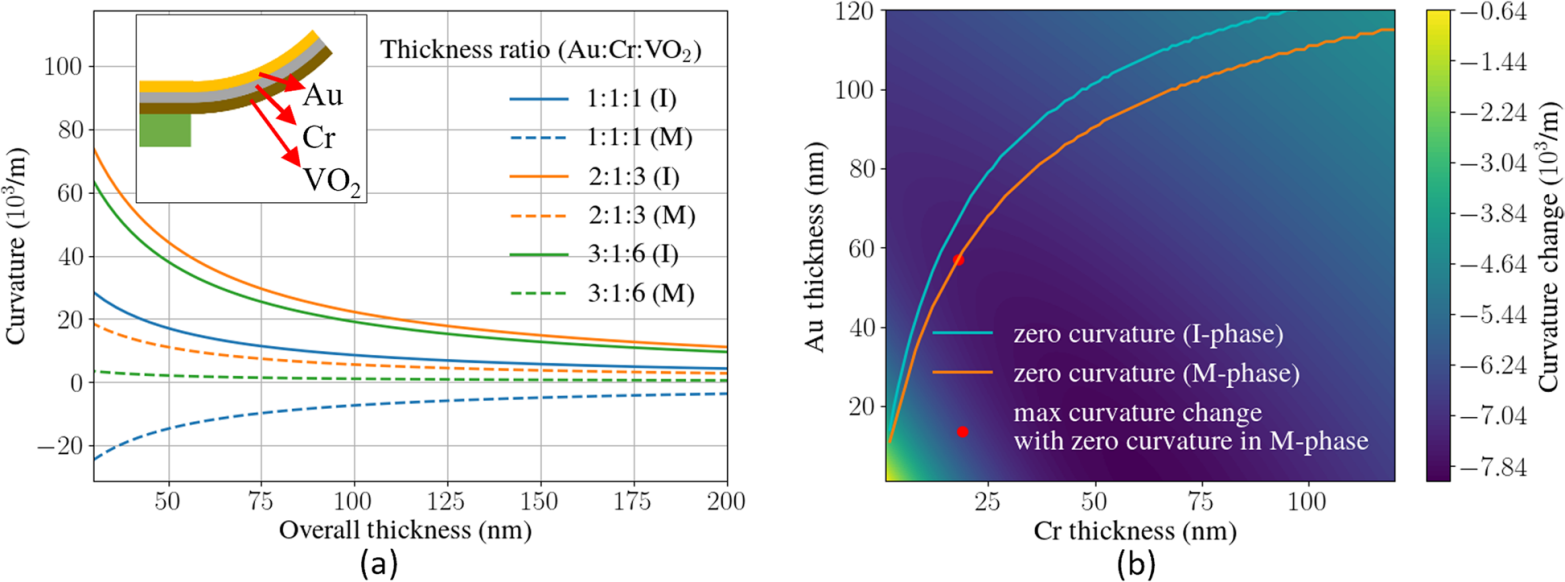
**a** Cantilever curvature vs. overall thickness for different Au/Cr/VO_2_ ratios. (I) and (M) indicate the VO_2_ in the insulating or metallic phases. Inset: Schematic of the tri-layer cantilever and the thin film materials of the cantilevers. **b** Cantilever curvature change during actuation for different Au/Cr thicknesses and 120 nm VO_2_. A deeper color indicates a larger curvature change against the VO_2_ phase transition. The blue line indicates 0 curvature when VO_2_ is in the insulating phase at 30 °C. The yellow line indicates 0 curvature when VO_2_ is in the metallic phase at 90 °C

To integrate VO_2_ as the actuation material, we employed a three-layer (gold-chromium-VO_2_) cantilever design, which provides high design flexibility and arbitrary control of curvatures [27] as shown in Fig. 2a inset. The curvature of a cantilever is determined by the combined residual stresses during the fabrication process in all three layers. When VO_2_ undergoes a phase transition, the volume shrinkage alters the internal stresses and leads to a change in the curvature of the cantilever.

To design the three-layer cantilever, we adapted the model by Nikishkov (Eqs. (1) − (3)) to calculate the curvature change before and after the VO_2_ phase transition [27, 69]. The curvature (*K*) of a cantilever is given by

1 $$\begin{array}{c}K=\frac{3{\sum }_{n=1}^{m}{E}_{n}^{\prime}{t}_{n}\left({y}_{n}+{y}_{n-1}-2{y}_{b}\right)\left(c-\left(1+{\nu }_{n}\right){\varepsilon }_{n}\right)}{2{\sum }_{n=1}^{m}{E}_{n}^{\mathrm{^{\prime}}}{t}_{n}\left[{y}_{n}^{2}+{y}_{n}{y}_{n-1}+{y}_{n-1}^{2}-3{y}_{b}\left({y}_{n}+{y}_{n-1}-{y}_{b}\right)\right]},\end{array}$$

2 $$\begin{array}{c}{y}_{b}=\frac{{\sum }_{n=1}^{m}{E}_{n}^{\mathrm{^{\prime}}}{t}_{n}\left({y}_{n}+{y}_{n-1}\right)}{2{\sum }_{n=1}^{m}{E}_{n}^{\mathrm{^{\prime}}}{t}_{n}},\end{array}$$

3 $$\begin{array}{c}c=\frac{{\sum }_{n=1}^{m}{E}_{n}^{\mathrm{^{\prime}}}{t}_{n}\left(1+{\upnu }_{n}\right){\upvarepsilon }_{n}}{{\sum }_{n=1}^{m}{E}_{n}^{\mathrm{^{\prime}}}{t}_{n}},\end{array}$$

where *E*_*n*_, *ν*_*n*_, *t*_*n*_, and *ε*_*n*_ are Young’s modulus, Poisson’s ratio, film thickness, and initial strain for the *n*th layer, respectively. And ${E}_{n}^{\prime}=\frac{{E}_{n}}{\left(1-{\nu }_{n}^{2}\right)}$, ${y}_{n}={y}_{n-1}+{t}_{n}$ and ${y}_{0}=0$. The Young’s modulus and Poisson’s ratio of the materials are available in Refs. [70, 71]. We first fabricated bi-layer cantilevers and measured their curvatures to verify the working principles and all the mechanical properties of the Au, Cr, and VO_2_ thin films that are required in the calculation [72]. With all these parameters, the curvature of a cantilever before and after the phase transition of VO_2_ could be predicted precisely. Using Eqs. (1) − (3), we find that the cantilever curvature is inversely proportional to the overall thickness for a fixed Au/Cr/VO_2_ thickness ratio, as shown in Fig. 2a. However, because our deposited VO_2_ thin films are polycrystalline, a thinner layer leads to a lower yield in fabrication. We find that using a 120 nm thick VO_2_ film could reach a balance between curvature change performance and engineering capability. Therefore, our design target is to maximize the curvature change while realizing zero curvature with metallic phase VO_2_.

Figure 2b shows the curvature change values before and after the phase transition of VO_2_ in cantilevers with a 120 nm thick VO_2_ layer and varying thicknesses of Au/Cr thin films. The blue and yellow lines indicate where zero curvature is realized with the insulating and metallic phases of VO_2_, respectively. The red dot on the yellow line corresponds to a layer thickness combination of 57 nm Au, 18 nm Cr, and 120 nm VO_2_, which provides the largest curvature change against the VO_2_ phase transition while maintaining zero curvature when VO_2_ is in the metallic phase. Therefore, this combination is used for device fabrication.

### Fabrication

The metamaterial was fabricated on a 650 µm thick double-side polished sapphire wafer. Atop the wafer, a SiO_2_ layer with a thickness of 500 nm was deposited via plasma-enhanced chemical vapor deposition. Afterwards, the VO_2_ layer was deposited using pulsed laser deposition. Photolithography with a laser writer was used to write the metamaterials pattern. The Cr and Au layers were sequentially deposited using an electron beam evaporator, followed by lift-off. The exposed VO_2_ was then etched using a fluorine-based inductively coupled plasma etcher to expose the SiO_2_ layer. The spiral cantilevers were then released using a 5% hydrofluoric acid wet etch, followed immediately by drying with a critical point dryer to ensure structural integrity.

**Fig. 3:**
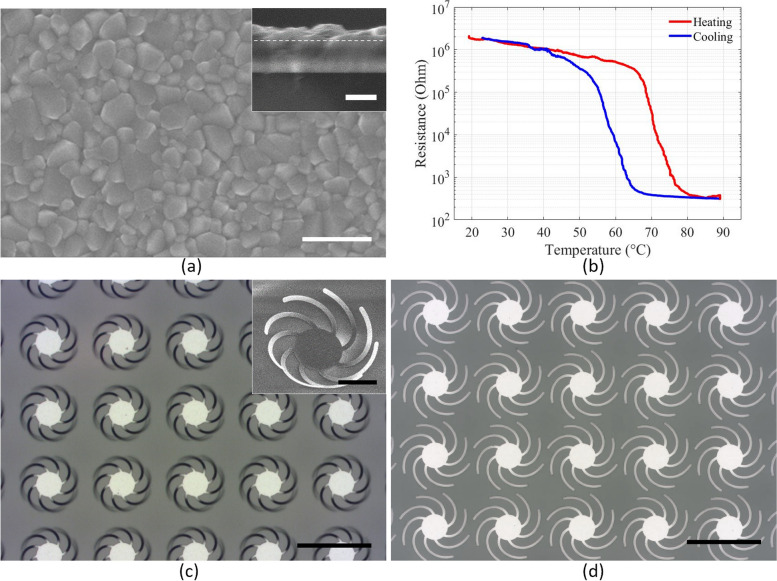
**a** SEM image of the VO_2_ thin film, the scale bar size is 500 nm. Inset: Cross-sectional view of ~ 120 nm VO_2_ on a 500 nm SiO_2_/Sapphire substrate, the dash line indicates the interface between VO_2_ and SiO_2_, the scale bar is 500 nm. **b** Resistance–temperature change of the VO_2_ thin film. **c** Optical microscope image of the spiral cantilevers curving up at 30 °C, the scale bar is 200 μm. Inset: SEM image of the spiral cantilevers curving up. The scale bar is 50 μm. **d** Optical microscope image of the spiral cantilevers fully flat at 90 °C, the scale bar is 200 μm

Multiple characterization methods were used to verify the quality of the deposited 120 nm thick VO_2_ thin films. As shown in the scanning electron microscope (SEM, Zeiss Merlin) image of Fig. 3a, the VO_2_ surface consists of densely packed, uniformly sized grains with a rounded polyhedral shape. The size of the grains is consistent across the surface, with no larger agglomerations or clustering, indicating a uniform thin film deposition. The inset SEM image is the cross-sectional view of the 120 nm thick VO_2_ thin film on a 500 nm SiO_2_/sapphire substrate.

The resistance of the VO_2_ thin film was measured as a function of temperature to verify the insulator–metal phase transition. As shown in Fig. 3b, the resistance decreases dramatically by more than three orders of magnitude when increasing the temperature from 20 °C to 90 °C, indicating a high-quality VO_2_ film.

Figure 3c and d are optical microscope images of the spiral cantilever metamaterials. At room temperature, the spiral cantilever arms curve up and form a chiral structure, as shown in Fig. 3c. The inset is a tilted SEM image of the spiral cantilever unit cell. At high temperatures, the VO_2_ undergoes the insulator–metal phase transition, and the spiral cantilever beams become fully flat, as in Fig. 3d.

### Polarization modulation of THz optical activity

**Fig. 4:**
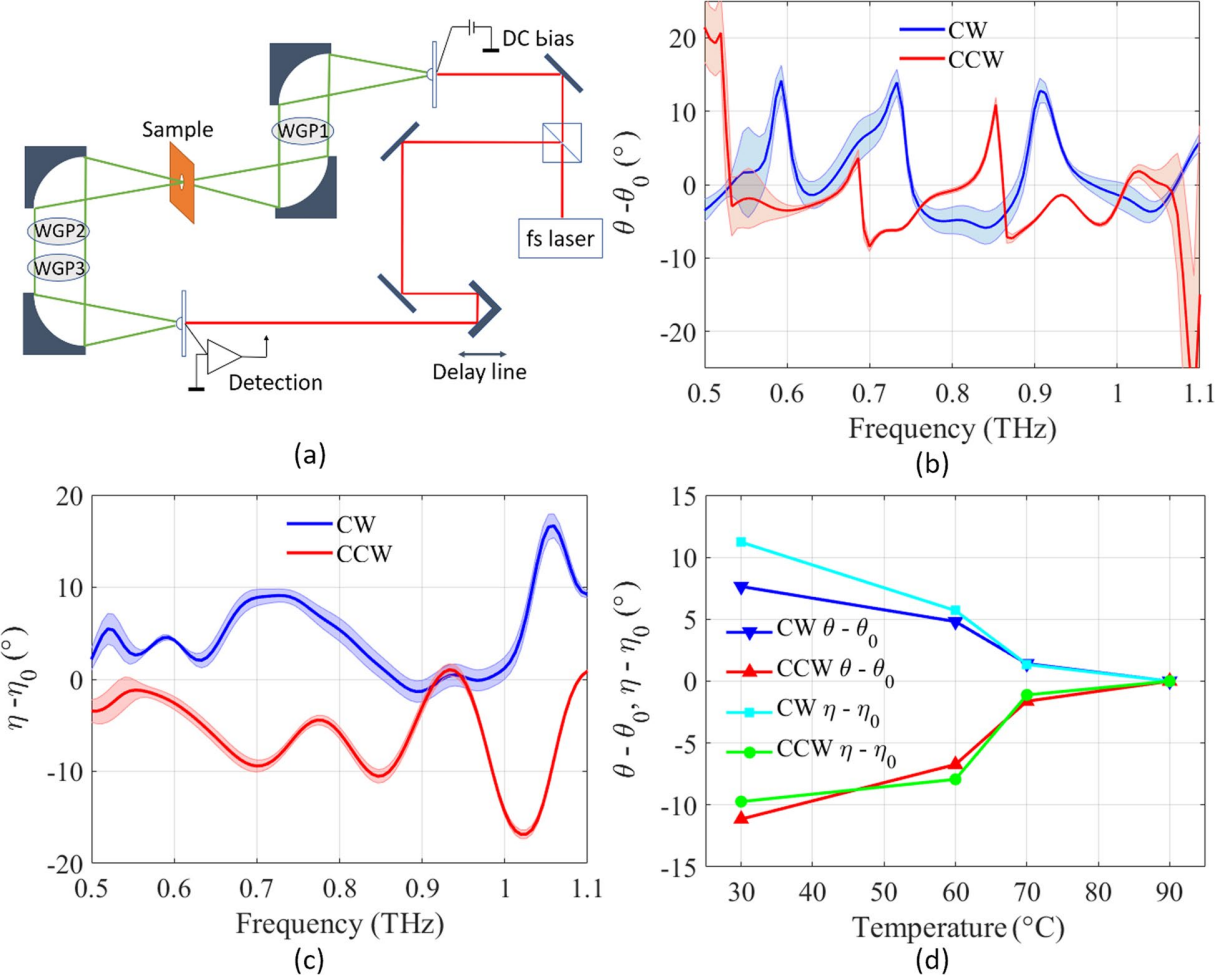
**a** Schematic of the THz-TDS system. **b** Measured azimuth polarization rotation angle changes between the curved state, *θ* (30 °C), and the flat state *θ*_0_ for clockwise and counterclockwise spirals. **c** Measured ellipticity angle changes between the curved state *η* (30 °C), and the flat state *η*_0_, for clockwise and counterclockwise spirals. **d** Measured azimuth polarization rotation angle change *θ–θ*_0_ and ellipticity angle change *η–η*_0_ vs. temperature at 0.72 THz

Optical activity modulation was studied using THz time-domain spectroscopy (THz-TDS), as shown in Fig. 4a. Three wire grid polarizers (WGP) are placed in the beam path. WGP1 is used to linearly polarize the incident wave in the *y*-direction. To detect the change in the polarization states, another WGP3 is used as a crossed analyzer detecting the x-component of the signal to obtain the vectoral information of the arbitrary polarized THz wave, WGP2 is needed with two orientations. With this method, WGP2 extracts the polarization components of the transmitted THz wave at –45° and + 45° with the horizontal (*x*) axis (*E*_1_(*t*) and *E*_2_(*t*), respectively) [73].

By collecting the time domain data as described in the Supplementary Information, azimuth rotation angle change *θ* and ellipticity angle change *η* can be calculated [73]. The corresponding results are presented in Fig. 4b, c, and d. Figure 4b plots the *θ* change, and Fig. 4c plots the *η* change before and after the phase transition of VO_2_ across a range of frequencies (0.5–1.1 THz). *θ*_0_ and *η*_0_ indicate the *θ* and *η* values measured at 90 °C, while *θ* and *η* indicate the values measured at other temperatures (30 °C in Fig. 4b and c). As the temperature rises from 30 °C to 90 °C, the spiral cantilever structure deforms from curved up to almost flat, and the *θ* values at different frequency ranges can be effectively modulated. Additionally, the ellipticity angle values indicate that the spiral cantilevers not only modified the direction of polarization but also converted the incoming linearly polarized THz waves into a partial circular polarization. In Fig. 4b and c, the error bars represent the uncertainty introduced by the potential misalignment of the three polarizers, each of which may deviate by up to ± 5° from its intended orientation. Considering these variations for all three polarizers, plots for both the polarization azimuthal rotation angle and the ellipticity angle are generated.

The mismatch between simulation and experimental results is likely from the modeling in COMSOL simulations. In COMSOL simulation, we built the structure with the same geometry as the real device but used a perfect electrical conductor (PEC) layer with zero thickness to represent the spiral cantilevers. Due to the severe scale difference between nanometer-thick layers and hundreds of micron-lengths in three dimensions, implanting real devices in COMSOL simulations causes an unaffordable number of meshing elements. Therefore, we must use the PEC model to perform an approximated simulation of the modulation principles. In addition, both fabrication and characterization will introduce slight variances sample by sample. The combined effect may cause some mismatches between the simulation and experimental results, and among devices.

Figure 4d shows the variation in azimuth rotation angle at 0.72 THz as the temperature is increased. As the temperature rises from room temperature to 90 °C, the measured *θ* and *η* values decrease correspondingly. A substantial 10° shift in *θ* and *η* are observed with the temperature change. Notably, a significant shift occurs when the temperature changes from 60 °C to 70 °C across the phase-transition of VO_2_, resulting in a change of more than 5° in both *θ* and *η* within only a 10 °C temperature difference. The extent of the *θ* and *η* modulations showcase the material’s high sensitivity to temperature changes. This is particularly noteworthy because it underscores the capability of the metamaterial to function as a versatile modulator across a broad spectrum of operational conditions.

The comparison with previous works in THz polarization modulation is summarized in Table S1 of the Supplementary Information. The modulation of *θ* and *η* (about 15°) achieved with our device is comparable with previous reports using high driving voltages (about 6°) [43] and pneumatic forces (about 28°) [39]. While providing a reasonably large modulation depth and broad operation wavelength range, our devices are fabricated by one-step photolithography and actuated only by a small temperature increase near room temperature.

## Conclusion

In conclusion, MEMS-actuated terahertz metamaterials for polarization modulation driven by phase-transition materials are demonstrated. By harnessing the phase-transition properties of the material VO_2_, our THz metamaterials exhibit efficient and broad-spectrum THz polarization modulation with a simple operational approach. These characteristics make it a promising candidate for diverse THz applications such as telecommunication, THz imaging, and biosensing. Fabricating the spiral cantilevers onto a suspended membrane may allow the cantilevers to flex in both upward and downward directions, facilitating bidirectional tuning, which is expected to significantly enhance the versatility of our device. In addition, by integrating a heating circuit directly onto the sample, we anticipate a marked improvement in modulation speed. This will enable faster response times, making our device more efficient and suitable for a broader range of applications.

### Supplementary Information

Supplementary Material 1.

## Data Availability

The data that support the findings of this study are available from the corresponding author, upon reasonable request.
