# Supplementary material for: MEMS-actuated terahertz metamaterials driven by phase-transition materials
Source: Front Optoelectron. 2024 May 27;17(1):13. doi: 10.1007/s12200-024-00116-4 (PMC11128424; doi:10.1007/s12200-024-00116-4)
Supplement: Supplementary file 1 — Supplementary Material 1. [file 12200_2024_116_MOESM1_ESM.pdf]

Supplementary Information

MEMS-actuated terahertz metamaterials driven by phase-transition materials

Zhixiang Huang[1], Weipeng Wu[2], Eric Herrmann[1], Ke Ma[1], Zizwe A. Chase[3], Thomas A. Searles[3], M. Benjamin Jungfleisch[2], Xi Wang[1]

Xi Wang

wangxi@udel.edu

1 Department of Materials Science and Engineering, College of Engineering, University of Delaware, Newark, DE 19716, USA

2 Department of Physics and Astronomy, College of Arts and Sciences, University of Delaware, Newark, DE 19716, USA

3 Department of Electrical and Computer Engineering, College of Engineering, University of Illinois Chicago, Chicago, IL 60607, USA

Methods

Device Fabrication and Configuration

Our devices are fabricated using standard nanofabrication techniques, including lithography, deposition, and etching. As shown in Fig. S1, first, a 500 nm thick layer of SiO_2 was deposited on 650 µm thick double-side polished sapphire substrates using plasma-enhanced chemical vapor deposition (PECVD, Plasma-Therm Versaline). After that, VO_2 was deposited using pulsed laser deposition (PVD products PLD-4000) with good uniformity of polycrystalline phase. The deposition temperature was 600 °C, and the O_2 concentration was set to 10 mTorr. After the deposition of about 120 nm thick VO_2 films, the samples were patterned using photolithography. Spiral patterns were directly written using a laser writer (Heidelberg MLA100 Maskless Aligner) and developed after exposure. An electron beam evaporator (PVD products UHV E-Beam System) was used to deposit 18 nm of Cr and 57 nm of Au on the samples, followed by liftoff to complete the patterning of the metal spiral patterns. We then employ inductively coupled plasma (F-ICP, Plasma-Therm Apex SLR) etching to etch down through the SiO_2 layer. The SF_6 gas selectively etches the VO_2/SiO_2, while Au served as a hard mask during the dry etching process. The SiO_2 layer underneath VO_2 was wet etched isotropically using 5% hydrofluoric acid (HF), following critical point drying to release the final Au/Cr/VO_2 cantilever structure.

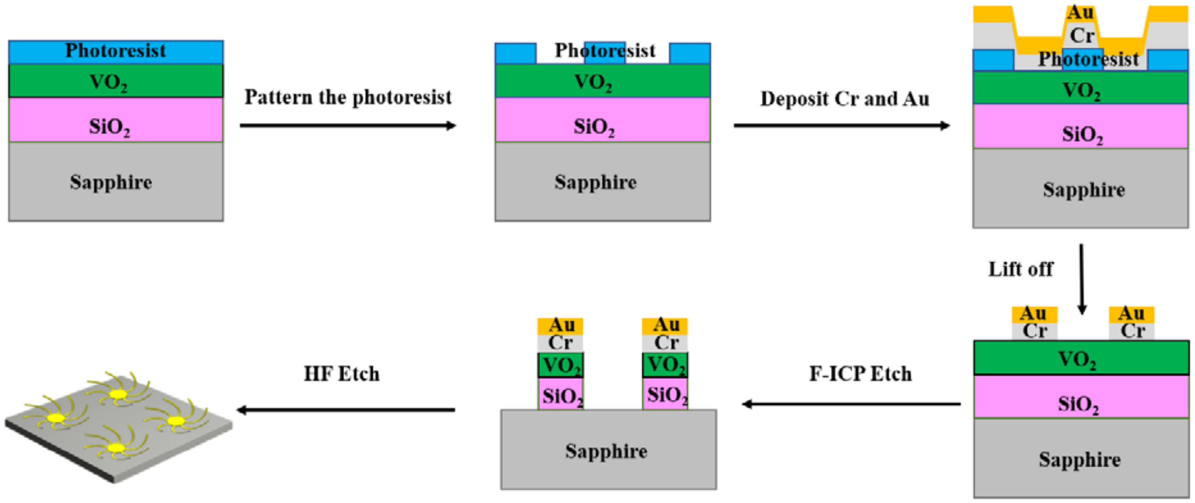

Fig. S1 Key fabrication procedures of the metamaterials.

Terahertz Time-domain Spectroscopy

The terahertz time-domain spectroscopy system was used to characterize the spiral cantilever metamaterial. The TDTS system that we utilize is depicted in Fig. S2. This system consists of a standard TDTS configuration along with three distinct polarizers. Three wire-grid polarizers were inserted to perform three distinct tests on the sample and three reference tests, using air as the reference medium, as illustrated in the bottom figures.

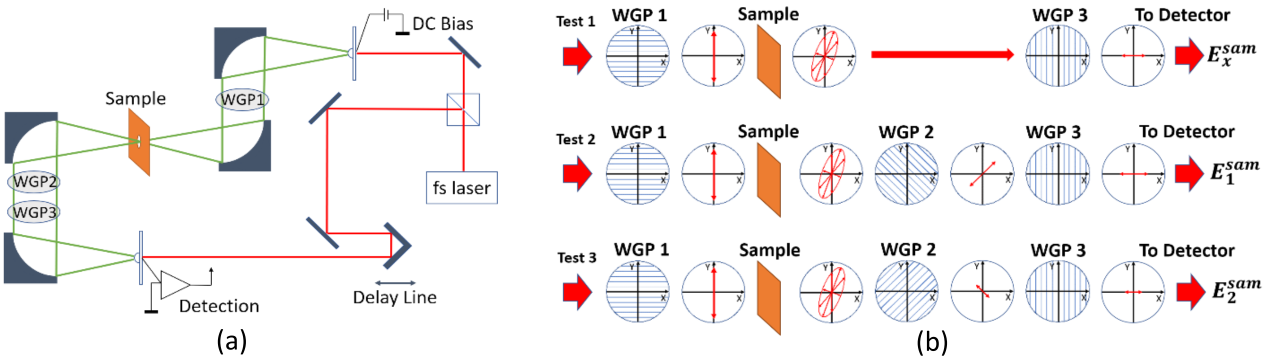

Fig. S2 (a) Schematic of the THz-TDS system. (b) Schematic of the three required measurements

Fig. S2(b) shows the three tests performed on the sample [1]. WGP1 and WGP3 were kept the same, while WGP2 was adjusted. In the first measurement, $E_x(t)$ can be measured. In the second test, WGP2 is set to -45° to get E_1^{sam} data. Then, in the third test, WGP2 is set to +45° to collect E_2^{sam} data. Then the phase and amplitude of the y-component of the transmitted THz wave $E_y(t)$ can be obtained by subtracting $E_2^{sam}(t)$ from $E_1^{sam}(t)$ (Eq. (S1)).

$$E_y^{sam}(t) = E_1^{sam}(t) - E_2^{sam}(t) \quad \text{(S1)}$$

In addition to this, the electric field of THz radiation without the sample (E^{ref}) was measured as the reference signal of the incident wave. The setups were the same except for the absence of the sample. Another three tests were performed to get E_x^{ref}, E_1^{ref}, and E_2^{ref}. Then, by subtracting E_2^{ref} from E_1^{ref}, the E_y^{ref} values were extracted.

After collecting all the data sets, which are E_x^{ref}, E_y^{ref}, E_x^{sam}, and E_y^{sam}, a Fourier transform was applied to the electric field in the time domain to obtain the electric field vectors in the frequency domain, denoted as $\tilde{E}^{sam}$ and $\tilde{E}^{ref}$. The frequency domain data maintains the original information of the amplitude and phase of the sample. By comparing $\tilde{E}^{sam}$ with $\tilde{E}^{ref}$, the complex Jones matrix of the samples was extracted. The relationship between the two vectors, $\tilde{E}^{sam}$ and $\tilde{E}^{ref}$, is defined by Eq. (*S*2). And $\tilde{T}$ is the Jones matrix of the sample.

$$\tilde{E}^{sam} = \tilde{T}\tilde{E}^{ref} \qquad \text{(S2)}$$

Given that the sample exhibits eight-fold rotational symmetry, this matrix is specified as $\tilde{T} = \begin{bmatrix} \tilde{t}_1 & \tilde{t}_2 \\ -\tilde{t}_2 & \tilde{t}_1 \end{bmatrix}$. This configuration ensures that the matrix remains invariant under any rotational operation, indicating that the rotation of polarization is independent of the incident wave's polarization orientation [2]. By analyzing the measured values of $\tilde{E}^{sam}$ and $\tilde{E}^{ref}$, solving Eq. (*S*3), we calculated the values of $\tilde{t}_1$ and $\tilde{t}_2$ at different frequencies.

$$\begin{bmatrix} \tilde{t}_1 \\ \tilde{t}_2 \end{bmatrix} = \begin{bmatrix} \tilde{E}_x^{ref} & \tilde{E}_y^{ref} \\ \tilde{E}_y^{ref} & -\tilde{E}_x^{ref} \end{bmatrix}^{-1} \begin{bmatrix} \tilde{E}_x^{sam} \\ \tilde{E}_y^{sam} \end{bmatrix} \qquad \text{(S3)}$$

The spectra for the polarization azimuth rotation and the ellipticity angles were derived from the Jones matrix using the equations defined in Eqs. (*S*4) (*S*5).

$$\theta = -\frac{1}{2}tan^{-1}\frac{2Re(\tilde{t}_1\tilde{t}_2^*)}{|\tilde{t}_1|^2 + |\tilde{t}_2|^2} \qquad \text{(S4)}$$

$$\eta = -\frac{1}{2}sin^{-1}\frac{2Im(\tilde{t}_1\tilde{t}_2^*)}{|\tilde{t}_1|^2 + |\tilde{t}_2|^2} \qquad \text{(S5)}$$

Simulation Details

COMSOL Multiphysics simulations were performed in a simulation space 200 µm-wide in the x and y directions and 800 µm-tall in the z direction. Periodic boundary conditions were applied in the x and y directions. A 650 µm-thick sapphire substrate was placed in the middle of the simulation domain. Periodic ports were used at boundaries in the z direction, with linearly polarized light excited from the sapphire side.

Spiral cantilevers were placed on the sapphire. As shown in Figure S3, the center of the spiral cantilevers is a circular plate with a 30-µm radius. Spiral arms extrude from the edge of the circular plate, with a width of 5 µm.

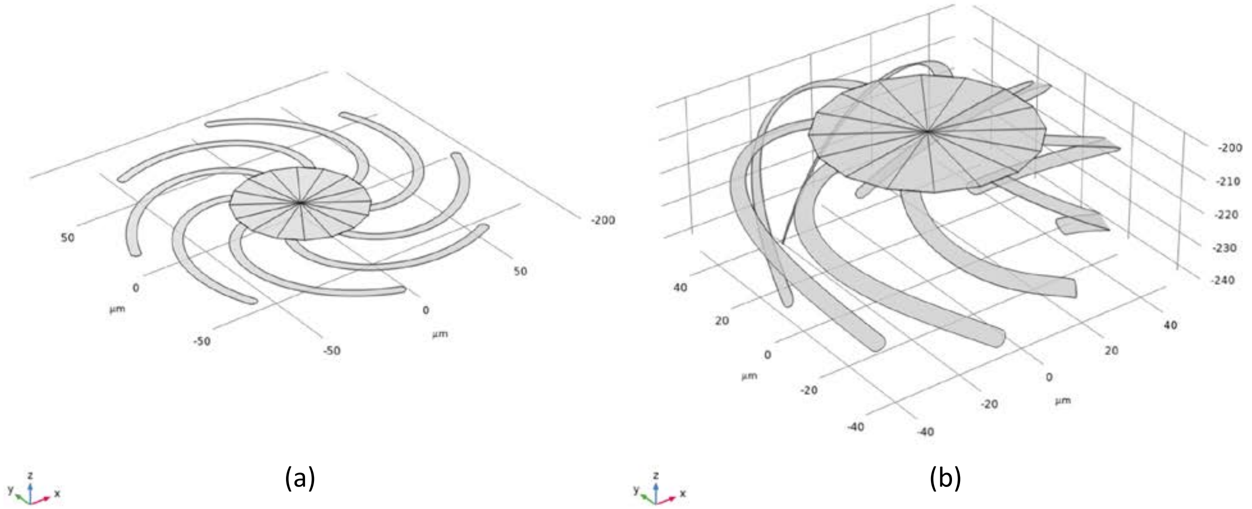

Fig. S3 (a) Schematic of flat CCW spiral cantilevers. (b) Schematic of curved CCW spiral cantilevers. The sapphire substrate was excluded from the schematic.

To build the spiral arms, we first defined a spiral spline by a modified constant speed spiral (Archimedean spiral).

For flat spiral arms, we defined:

$$X = v \cdot t \cdot cos(w \cdot t)$$

$$Y = 0.5 \cdot v \cdot t \cdot sin(w \cdot t)$$

$$Z = 0$$

where *v* is the linear velocity, *w* is the angular velocity, and *t* is time.

For curved spiral arms, we defined:

$$X = v \cdot t \cdot cos(w \cdot t)$$

$$Y = 0.5 \cdot v \cdot t \cdot sin(w \cdot t)$$

$$Z = -(r - \sqrt{r^2 - (t \cdot v)^2})$$

where *r* is the inverse of the curvature of the spiral arm. The y-direction has a slower growing speed ($0.5 \cdot v$) than the flat case (v). This is to better approximate the 3D structure of the spirals curved along its main direction. We found that with these parameters, the structure created matches our fabricated prototype well.

After creating an individual spiral spline, it was shifted 30 μm along x-direction to have its starting point move from the center to the edge of the circle. One spiral arm was created by sweeping along the spiral spline. Then, the spiral arm was rotated and copied along the z-axis placed at the center of the circular plate. Considering the symmetry, we used eight cantilever arms (45° rotation), which provides an optimal compromise, maximizing the efficiency within practical manufacturing yield. After further optimization, we found that *v* = 100 μm/s, *w* = 0.7π rad/s, *t* = [0, 0.8] sec, *r* = 140 μm could provide a high modulation depth while keeping spiral arms away from each other at both flat and curved status. After building the spiral cantilevers, we set them as the perfect electric conductor layer for simulations.

Comparison with Previous Works

Table S1 Comparison with previous works in THz polarization modulation of the azimuth rotation angle θ and the ellipticity angle η.

Design	Highest Modulation Depth	Operation Wavelength Range	Operation Method
Deformable MEMS spiral metamaterial with enantiomeric switching of chirality [3]	About 28.1° changes in azimuth rotation angle θ and ellipticity angle η	0.4-1.8 THz	pneumatic force with a sealed gas chamber
Patterned plasmonic structure on parylene sheets with periodic kirigami cuts [4]	About 15° changes in azimuth rotation angle θ and ellipticity angle η with maximum values of 80° and 40° at one resonant wavelength	0.2-1.6 THz	Stress applied by a mechanical stage
Single-layer chiral metamaterial structure with electrostatically gated graphene [5]	A maximum of 20° change of azimuth rotation angle θ at a resonant frequency (1.75 THz)	1.4 – 2.5 THz	Backgated voltage > 100 V
Conjugated double Z metamaterial with a graphene layer [6]	Up to 10° change of azimuth rotation angle θ off-resonance	0.6-1.6 THz	5 V ion gel gating
three-rotating-layer metallic grating structure [7]	Tunable azimuth rotation angle from –90° to 90°.	0.1-0.6 THz	Mechanically rotating the three composite grating layers
Chiral metamolecules incorporated with a photoactive medium (Si) [8]	About 10° change in azimuth rotation angle θ	0.84-1.07 THz	Photoexcitation stimuli. 1 $mJ\cdot cm^{-2}$. Fabrication involving electroplating.
a three-dimensional helical spiral metamaterial [9]	About 6° change in azimuth rotation angle θ and 4° change in ellipticity η	0.4-2.3 THz	electrostatic force with 350 V bias
single-layer microcantilevers metasurface with anisotropic response [10]	About 45° change in ellipticity at one specific frequency 0.81 THz	Near 0.81THz	electrostatic force with 40 V bias
Liquid crystal cell with metal wire grid and porous graphene as electrode [11]	About 90° change in azimuth rotation angle θ	0.5-2.5THz	Liquid crystal with 50V gate voltage
This work	About 15° changes in azimuth rotation angle θ and ellipticity angle η	0.5-1.1 THz	Heating stage near room temperature

References

1. N. Kanda, K. Konishi and M. Kuwata-Gonokami: Terahertz wave polarization rotation with double layered metal grating of complimentary chiral patterns. *Optics Express*, 15(18), 11117-11125 (2007) doi:https://doi.org/10.1364/OE.15.011117
2. L. Cong, N. Xu, W. Zhang and R. Singh: Polarization Control in Terahertz Metasurfaces with the Lowest Order Rotational Symmetry. *Advanced Optical Materials*, 3(9), 1176-1183 (2015) doi:https://doi.org/10.1002/adom.201500100
3. T. Kan, A. Isozaki, N. Kanda, N. Nemoto, K. Konishi, H. Takahashi, M. Kuwata-Gonokami, K. Matsumoto and I. Shimoyama: Enantiomeric switching of chiral metamaterial for terahertz polarization modulation employing vertically deformable MEMS spirals. *Nature Communications*, 6, 8422 (2015) doi:https://doi.org/10.1038/ncomms9422
4. W. Wu, S. Lendinez, M. T. Kaffash, R. D. Schaller, H. Wen and M. B. Jungfleisch: Controlling polarization of spintronic THz emitter by remanent magnetization texture. *Applied Physics Letters*, 121(5), 052401 (2022) doi:https://doi.org/10.1063/5.0096252
5. S. J. Kindness, N. W. Almond, W. Michailow, B. Wei, L. A. Jakob, K. Delfanazari, P. Braeuninger-Weimer, S. Hofmann, H. E. Beere, D. A. Ritchie and R. Degl'Innocenti: Graphene-Integrated Metamaterial Device for All-Electrical Polarization Control of Terahertz Quantum Cascade Lasers. *ACS Photonics*, 6(6), 1547-1555 (2019) doi:https://doi.org/10.1021/acsphotonics.9b00411
6. T.-T. Kim, S. S. Oh, H.-D. Kim, H. S. Park, O. Hess, B. Min and S. Zhang: Electrical access to critical coupling of circularly polarized waves in graphene chiral metamaterials. *Science Advances*, 3(9), e1701377 (2017) doi:https://doi.org/10.1126/sciadv.1701377
7. R.-H. Fan, Y. Zhou, X.-P. Ren, R.-W. Peng, S.-C. Jiang, D.-H. Xu, X. Xiong, X.-R. Huang and M. Wang: Freely Tunable Broadband Polarization Rotator for Terahertz Waves. *Advanced Materials*, 27(7), 1201-1206 (2015) doi:https://doi.org/10.1002/adma.201404981
8. D. Kong, X. Wu, B. Wang, T. Nie, M. Xiao, C. Pandey, Y. Gao, L. Wen, W. Zhao, C. Ruan, J. Miao, Y. Li and L. Wang: Broadband Spintronic Terahertz Emitter with Magnetic-Field Manipulated Polarizations. *Advanced Optical Materials*, 7(20), 1900487 (2019) doi:https://doi.org/10.1002/adom.201900487
9. T. Kan, A. Isozaki, N. Kanda, N. Nemoto, K. Konishi, M. Kuwata-Gonokami, K. Matsumoto and I. Shimoyama: Spiral metamaterial for active tuning of optical activity. *Applied Physics Letters*, 102(22), 221906 (2013) doi:https://doi.org/10.1063/1.4809533
10. X. Zhao, J. Schalch, J. Zhang, H. R. Seren, G. Duan, R. D. Averitt and X. Zhang: Electromechanically tunable metasurface transmission waveplate at terahertz frequencies. *Optica*, 5(3), 303-310 (2018) doi:https://doi.org/10.1364/OPTICA.5.000303
11. L. Wang, X.-W. Lin, W. Hu, G.-H. Shao, P. Chen, L.-J. Liang, B.-B. Jin, P.-H. Wu, H. Qian, Y.-N. Lu, X. Liang, Z.-G. Zheng and Y.-Q. Lu: Broadband tunable liquid crystal terahertz waveplates driven with porous graphene electrodes. *Light: Science & Applications*, 4(2), e253-e253 (2015) doi:https://doi.org/10.1038/lsa.2015.26
